# Modeling Analysis of Signal Sensitivity and Specificity by *Vibrio fischeri* LuxR Variants

**DOI:** 10.1371/journal.pone.0126474

**Published:** 2015-05-11

**Authors:** Deanna M. Colton, Eric V. Stabb, Stephen J. Hagen

**Affiliations:** 1 Department of Microbiology, University of Georgia, Athens, GA, United States of America; 2 Physics Department, University of Florida, Gainesville, FL, United States of America; Ghent University, BELGIUM

## Abstract

The LuxR protein of the bacterium *Vibrio fischeri* belongs to a family of transcriptional activators that underlie pheromone-mediated signaling by responding to acyl-homoserine lactones (-HSLs) or related molecules. *V*. *fischeri* produces two acyl-HSLs, *N*-3-oxo-hexanoyl-HSL (3OC6-HSL) and *N*-octanoyl-HSL (C8-HSL), each of which interact with LuxR to facilitate its binding to a “*lux* box” DNA sequence, thereby enabling LuxR to activate transcription of the *lux* operon responsible for bioluminescence. We have investigated the HSL sensitivity of four different variants of *V*. *fischeri* LuxR: two derived from wild-type strains ES114 and MJ1, and two derivatives of LuxR^MJ1^ generated by directed evolution. For each LuxR variant, we measured the bioluminescence induced by combinations of C8-HSL and 3OC6-HSL. We fit these data to a model in which the two HSLs compete with each other to form multimeric LuxR complexes that directly interact with *lux* to activate bioluminescence. The model reproduces the observed effects of HSL combinations on the bioluminescence responses directed by LuxR variants, including competition and non-monotonic responses to C8-HSL and 3OC6-HSL. The analysis yields robust estimates for the underlying dissociation constants and cooperativities (Hill coefficients) of the LuxR-HSL complexes and their affinities for the *lux* box. It also reveals significant differences in the affinities of LuxR^MJ1^ and LuxR^ES114^ for 3OC6-HSL. Further, LuxR^MJ1^ and LuxR^ES114^ differed sharply from LuxRs retrieved by directed evolution in the cooperativity of LuxR-HSL complex formation and the affinity of these complexes for *lux*. These results show how computational modeling of *in vivo* experimental data can provide insight into the mechanistic consequences of directed evolution.

## Introduction

Bacterial pheromone signaling was discovered in *Vibrio fischeri* [1], a bioluminescent symbiont that remains a model for LuxI/LuxR-type acyl-homoserine lactone (-HSL) systems. These systems are widespread among the Proteobacteria [2,3]. LuxI generates *N*-3-oxo-hexanoyl-HSL (3OC6-HSL), a membrane-permeable pheromone (also called an “autoinducer”) that can signal between cells [4,5]. At a sufficient concentration, 3OC6-HSL combines with LuxR to form multimers that bind to a “*lux* box” DNA sequence and activate transcription [6–8]. 3OC6-HSL-LuxR complexes bind to the *lux* box between the divergent *luxR* and *luxI* genes, activating *luxICDABEG* transcription and bioluminescence. The AinS/AinR system [9], which has fewer known homologs than (and bears no resemblance to) LuxI/LuxR, provides additional upstream control of luminescence. AinS generates *N*-octanoyl-HSL (C8-HSL) [10,11], which is sensed by AinR, thereby modulating a multi-component regulatory cascade that ultimately influences transcription of *luxR* [12,13]. Therefore the luminescence system synthesizes and responds to two HSL signals, C8-HSL and 3OC6-HSL. Although C8-HSL is a weaker activator of LuxR than 3OC6-HSL, both HSLs can bind LuxR directly [14].

LuxR homologs in various bacteria have evolved to respond to diverse cognate signals and modulate responses in different ways [15]. Even among *V*. *fischeri* there is a great deal of variability in luminescence, pheromone signaling, and LuxR sequences [16–18]. The two most-studied strains of *V*. *fischeri*, MJ1 and ES114, share the same core luminescence circuitry but their luminescence and signaling systems differ in relative output [19–21]. The luminescence of MJ1 is much brighter than that of ES114, and in broth cultures MJ1 accumulated μM quantities of 3OC6-HSL, over a thousand-fold more than did ES114 [20,22]. By contrast, ES114 generated five times more C8-HSL than did MJ1 [22]. Although most orthologs in the MJ1 and ES114 strains share 94–100% amino acid identity, their LuxR proteins are only 75% identical, suggesting divergent evolution of LuxR toward distinct function(s) in the two strains [16]. The functional and phenotypic differences that result from the variation of LuxR between *V*. *fischeri* strains remain to be investigated.

Functional divergence of LuxR has been demonstrated by “directed evolution”, which identified changes in LuxR that affect HSL sensitivity and specificity. Briefly, *luxR* from MJ1 was mutagenized and placed in a system that allowed sorting and screening for alleles that altered responses to various acyl-HSLs, particularly the non-cognate C8-HSL signal [23–25]. Second-generation LuxR derivatives were then generated by shuffling alleles. Interestingly, many of the second-generation derivatives that were most responsive to C8-HSL contained a T33A amino acid substitution that is naturally present in the LuxR of ES114 (LuxR^ES114^) [16,23]. Starting from a LuxR with T33A, S116A, and M135I variations (hereafter referred to as LuxR^A^), additional screening uncovered another variant (LuxR^B^) with an additional R67M change that virtually eliminated responsiveness to the cognate signal 3OC6-HSL but retained responsiveness to C8-HSL [24]. By comparing the LuxR sequence to the crystal structure of the related TraR protein, the authors concluded that the changes altering responsiveness to specific acyl-HSLs were, most likely, not all located in the effector-binding pocket [23]. LuxR responsiveness to an acyl-HSL requires not only binding of the signal, but also an effector-induced conformational change that influences multimerization and binding to the *lux* box; it remains unclear which of these phenomena were affected by directed evolution.

**Fig 1:**
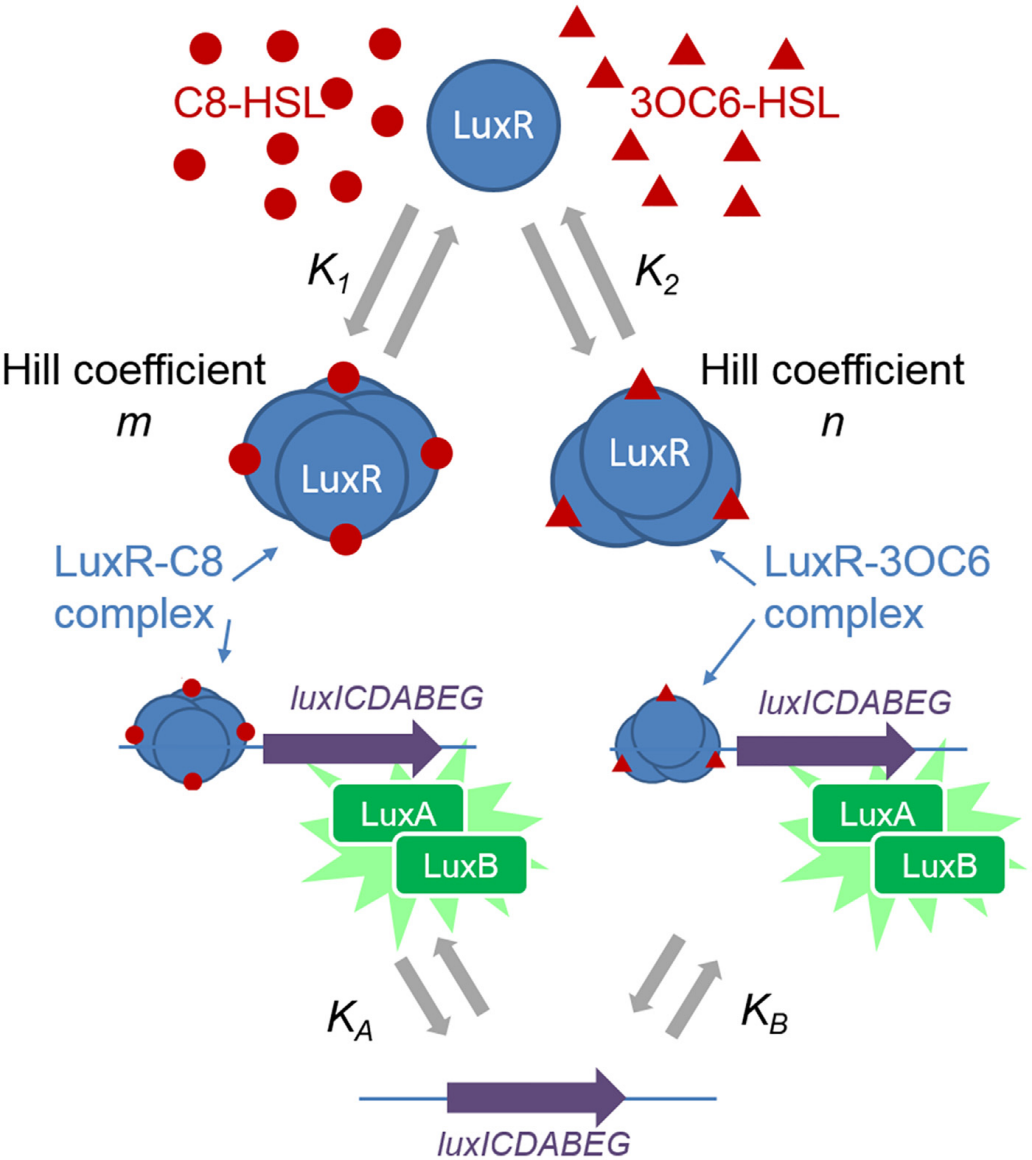
Model for LuxR-mediated induction of *V*. *fischeri* luminescence by C8-HSL and 3OC6-HSL. The pheromones C8-HSL and 3OC6-HSL interact with LuxR to form complexes with dissociation constants *K*_*1*_ and *K*_*2*_ (and Hill coefficients *m* and *n*) respectively. These complexes bind to the *lux* box (dissociation constants *K*_*A*_ and *K*_*B*_ respectively) to activate expression of the *lux* operon and synthesis of LuxA and LuxB, the subunits of the bacterial luciferase. The luminescence is proportional to the concentration of the LuxA-LuxB heterodimer [29].

Computational modeling of *in vivo* phenotypic data provides one approach to understanding the function of regulatory networks [26], and several prior authors have used network modeling techniques to analyze and interpret LuxI/LuxR control of luminescence in *V*. *fischeri* [27–33]. Here we use a modeling approach to trace the relationship between the HSL response of LuxR derivatives and interactions within the LuxI/LuxR network. We show that a minimal model for LuxR’s interactions with HSLs and the *lux* box (Fig 1) captures the diverse luminescence behavior evidenced by four LuxR derivatives over a wide range of 3OC6-HSL and C8-HSL concentrations. Fitting the model to experimental data on several different LuxRs provides insight into the differences between MJ1 and ES114 in their response to endogenous 3OC6-HSL and C8-HSL signals. It also shows quantitatively how the mutations discovered through directed evolution alter LuxR’s interactions with the two HSLs to modulate signal specificity and sensitivity.

## Results

**Fig 2:**
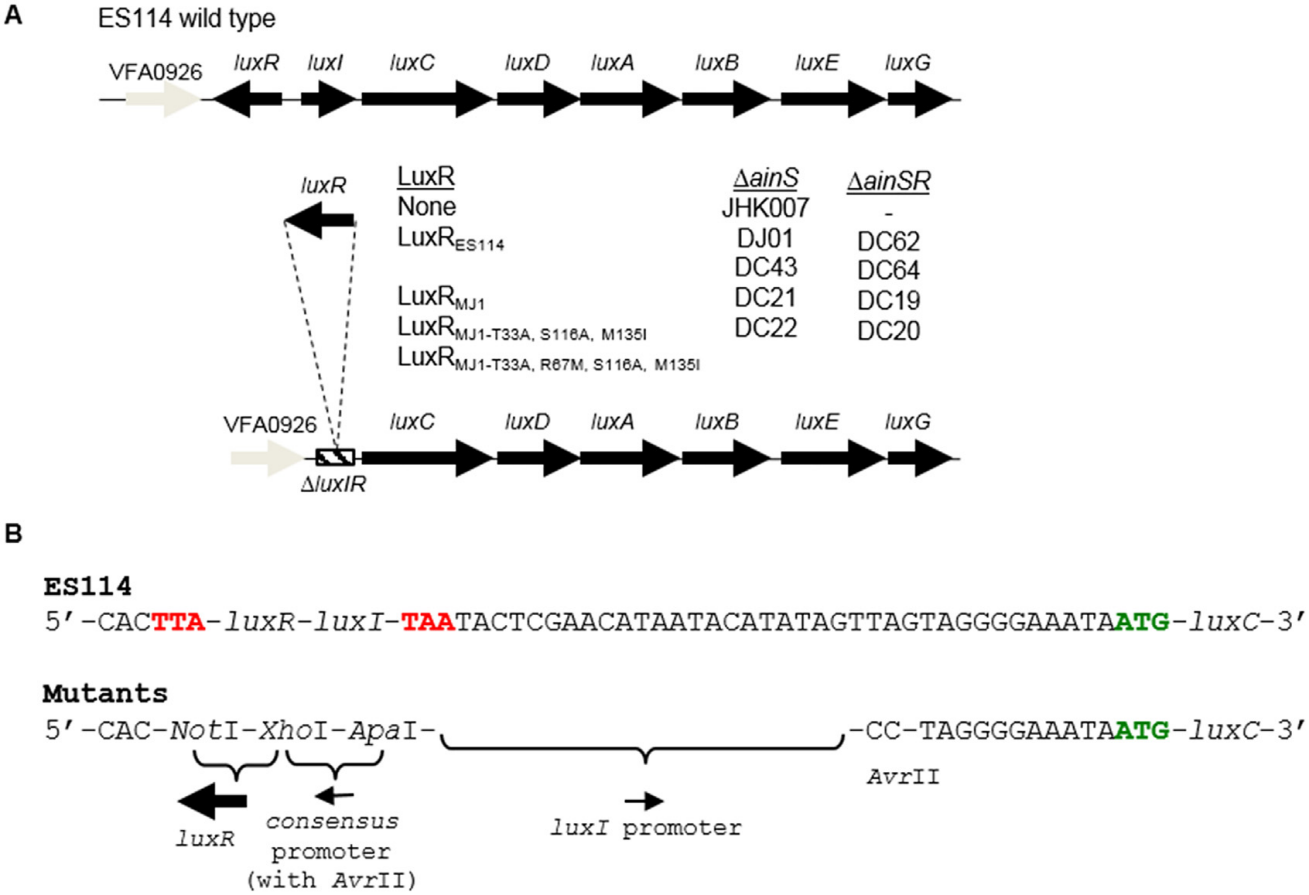
The *lux* operon in *V*. *fischeri* and genomic organization of strains engineered for this study. Panel (*A*) illustrates the genetic structure of the *lux* locus in parental wild-type strain ES114 (top) as well as the strains used to assay LuxR activity, wherein the native *luxRI* is deleted and *luxR* alleles are placed in an engineered construct between ORF VFA0926 and *luxC* (hatched box). Panel (*B*) shows specific sequences of ES114 aligned with those of engineered strains. Red sequences are stop codons for *luxR* (reverse strand) and *luxI*. The green ATG represents the start codon for *luxC*. Further details (e.g. cloning strategy, sequence of the “consensus promoter”, etc.) are provided in *Methods*.

In order to assess the responses of LuxR variants to defined combinations of 3OC6-HSL and C8-HSL inputs, we first engineered *V*. *fischeri* to remove potentially complicating factors. By conducting these studies in *V*. *fischeri*, we were able to evaluate LuxR function in the biophysical environment in which it evolved, e.g., with the cytoplasmic osmolytes of this marine bacterium and a chromosomal target promoter bearing native chromatin conformation. Table 1 lists the mutant *V*. *fischeri* strains that we generated, and Fig 2 illustrates their genotypes with respect to the *lux* locus. Here we give a brief description and rationale for the construction of these strains. In each of our test strains we deleted the *luxI* and *ainS* genes encoding HSL synthases. These deletions prevent endogenous production of 3OC6-HSL and C8-HSL respectively and allow us to control signal concentrations in the extracellular medium. Because native *luxR* is transcriptionally controlled by several regulators and is itself subject to pheromone-dependent regulation, we deleted the native *luxR* along with *luxI*. However, in doing so we also placed a *luxR* variant of our choosing back in the *lux* locus under control of a constitutive transcriptional promoter (Fig 2).

**Table 1:** Bacterial strains and plasmids used in this study.

Strain and plasmid	Relevant characteristics^*a*^	Source or reference
***E*. *coli***		
DH5α	φ80d*lacZ*ΔM15 Δ(*lacZYA-argF*)U169 *deoR supE44 hsdR17 recA1 endA1 gyrA96 thi-1 relA1*	[41]
DH5αλ*pir*	DH5α lysogenized with λ*pir*	[42]
CC118λ*pir*	***Δ***(*ara-leu*) *araD* ***Δ*** *lac74 galE galK phoA20 thi-1 rpsE rpsB argE*(Am) *recA* λ*pir*	[47]
***V*. *fischeri***		
DC19	ES114; Δ *ainSR* Δ *luxR-luxI*, mutant *luxR* ^*A*^ (MJ1-T33A, S116A, M135I), P_*luxI*_-*luxCDABEG*	This study
DC20	ES114 Δ *ainSR* Δ *luxR-luxI*, mutant *luxR* ^*B*^ (MJ1-T33A, R67M, S116A, M135I), P_*luxI*_-*luxCDABEG*	This study
DC21	ES114 Δ *ainS* Δ *luxR-luxI*, mutant *luxR* ^*A*^ (MJ1-T33A, S116A, M135I), P_*luxI*_-*luxCDABEG*	This study
DC22	ES114 Δ *ainS* Δ *luxR-luxI*, mutant *luxR* ^*B*^ (MJ1-T33A, R67M, S116A, M135I), P_*luxI*_-*luxCDABEG*	[48]
DC36	ES114 Δ *ainS lacI* ^q^ P_*A1/34*_-*luxCDABEG*	This study
DC43	ES114 Δ *ainS* Δ *luxR-luxI*, *luxR* ^*MJ1*^, P_*luxI*_-*luxCDABEG*	This study
DC44	ES114 Δ *ainSR lacI* ^q^ P_*A1/34*_-*luxCDABEG*	This study
DC62	ES114 Δ *ainSR* Δ *luxR-luxI*, *luxR* ^*ES114*^, P_*luxI*_-*luxCDABEG*	This study
DC64	ES114 Δ *ainSR* Δ *luxR-luxI*, *luxR* ^*MJ1*^, P_*luxI*_-*luxCDABEG*	This study
DJ01	ES114 Δ *ainS* Δ *luxR-luxI*, *luxR* ^*ES114*^, P_*luxI*_-*luxCDABEG*	This study
ES114	Wild-type isolate from *E*. *scolopes*	[19]
EVS102	ES114 Δ *luxCDABEG*	[34]
JB22	ES114 *lacI* ^q^ P_*A1/34*_-*luxCDABEG*	[34]
JHK007	ES114 Δ *ainS* Δ *luxR-luxI*, P_*luxI*_-*luxCDABEG*	This study
NL55	ES114 Δ *ainSR*	[49]
NL60	ES114 Δ *ainS*	[48]
**Plasmids** ^***b***^		
pDC36	Δ *luxRI* replacement LuxR-dependent bioreporter; *luxR* ^A^ (encoding MJ1 LuxR variant T33A S116A M135I)	This study
pDC37	Δ *luxRI* replacement LuxR-dependent bioreporter; *luxR* ^B^ (encoding MJ1 LuxR variant T33A R67M S116A M135I)	This study
pDC44	Δ *luxRI* replacement LuxR-dependent bioreporter; *luxR* ^-^	This study
pDC55	Δ *luxRI* replacement LuxR-dependent bioreporter; *luxR* ^MJ1^	This study
pDJ01	Δ *luxRI* replacement LuxR-dependent bioreporter; *luxR* ^ES114^	This study
pEVS104	conjugative helper plasmid; R6Kγ, *oriT*_RP4_, *kanR*	[46]
pJLB72	*luxR*-*luxI*- multiple cloning site-*luxC*, ColE1, R6Kγ, *oriT*_RP4_, *kanR*, *camR*	[34]
pJLB101	*lacI* ^q^ P_*A1/34*_-*luxCDABEG*, ColEI, R6Kγ, *oriT*_RP4_, *kanR*, *camR*	[34]
pLuxR-G2E	p15A *kanR*; *luxR* ^A^ (MJ1 LuxR-T33A S116A M135I)	[23]
pLuxR-G2E-R67M	p15A *kanR*; *luxR* ^B^ (MJ1 LuxR-T33A R67M S116A M135I)	[24]
**Oligonucleotides** ^***c***^		
5’-LuxRXhoI	CGA ACG GCT CGA GCA TGA AAA ACA TAA ATG CCG ACG ACA C	This study
3’-LuxRNotI	CGT TCG CGC GGC CGC CGT ACT TAA TTT TTA AAG TAT GGG CAA TC	This study
5’-ESll4luxRXhoI	CGA ACG CTC GAG ATG AAC ATT AAA AAT ATA AAT GCT AAT GAG AAG ATA ATT G	This study
3’-ES114luxRNotI	CGT TCG GCG GCC GCT TAA TTT TTA AGG TAT GGA CAA TTA ATG G	This study
Pconoligo1	CTT GAC ATA AAG TCT AAC CTA GGG TAT AAT C	This study
Pconoligo2	TCG AGA TTA TAG GGT AGG TTA GAC TTT ATG TCA AGG GCC	This study
PluxIF2	GTA GGG CCC GGA AAC GTG GTG TTA ACA TTG C	This study
PluxIR	GCT CCT AGG CAT TAC AGC CAT GCA ACC TCT C	This study
luxRdnFNotI	TTA GCG GCC GCG TGT ATG AAT AAA ACT TTA TGC CTA TAG	This study
luxRdnRNheI	TTA GCT AGC GCT GCC AAT ACC GAC TTT ACG TGC TTT ATC	This study

^*a*^ Drug resistance abbreviations used: *camR*, chloramphenicol resistance (*cat*); *ermR*, erythromycin resistance; *kanR*, kanamycin resistance

^*b*^ All alleles cloned in this study are from *V*. *fischeri* strain ES114. Replication origin(s) of each vector are listed as R6Kγ, p15A, or ColE1.

^*c*^ All oligonucleotides are shown 5’ to 3’. Underlined regions highlight restriction-enzyme recognition sites.

Next, although *luxR* was uncoupled from transcriptional control by the Ain system [12], we considered the possibility that the C8-HSL-dependent receptor AinR might confound our results, for example by binding and titrating C8-HSL or by modulating luminescence through another mechanism. We therefore produced strains in which *ainR* was deleted along with *ainS*.

Finally, we used control strains to test our experimental setup. First, we generated strain JHK007, which is isogenic to the test strains but lacks a *luxR* variant (Fig 2A). JHK007 produces little or no detectable bioluminescence, with background determined using the dark Δ *luxCDABEG* mutant EVS102. More importantly, JHK007 does not induce bioluminescence in response to 3OC6-HSL or C8-HSL (data not shown). We also tested the luminescence of strain JB22, wherein *luxCDABEG* is controlled by isopropyl-β-D-thiogalactoside (IPTG) from a non-native promoter [34]. We found that when induced by IPTG, under the conditions of our assays, JB22 is brighter than any of the strain and HSL combinations below (data not shown). Thus, luminescence output does not appear to be limited by the conditions (e.g., temperature and oxygen) in the plate reader where optical density and luminescence measurements were taken.

### Luminescence response to HSLs

**Fig 3:**
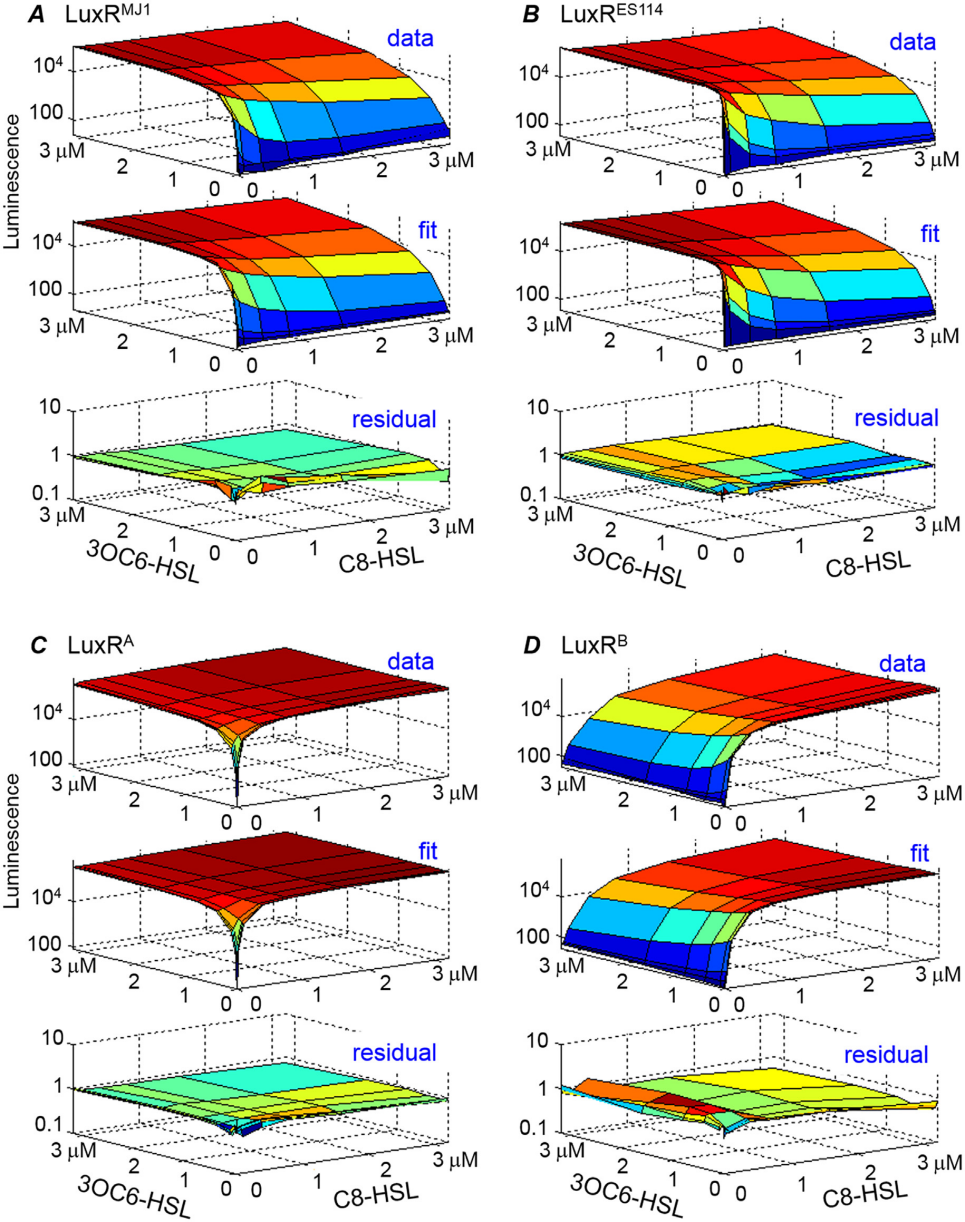
Comparing data and fit for Δ*ainRS* mutants of the four LuxR variants. Each of panels (*A*)-(*D*) shows a representative luminescence dataset and fit for one of the Δ*ainRS* strains, where luminescence is measured as a function of C8-HSL and 3OC6-HSL concentration. The luminescence (vertical) axis for data is shown on a logarithmic scale. The lower figure of each group shows the residual on a logarithmic scale, *i*.*e*. the ratio *data/fit* is shown on a logarithmic scale. The set of parameter values obtained in 150 fits of 3 datasets for each LuxR are shown in Fig 6 and summarized in Table 2.

Fig 3 and S1 Fig show that the luminescence of each strain increases in response to one of the 3OC6-HSL and C8-HSL signals, although the particular responses of the LuxR variants differ qualitatively. The luminescence of strains expressing LuxR^MJ1^ and LuxR^ES114^ is strongly activated by 3OC6-HSL, but their luminescence is weakly (if at all) activated by C8-HSL. The LuxR^B^ strain shows the opposite behavior, activated strongly by C8-HSL but not 3OC6-HSL. The LuxR^A^ strain is almost equally activated by both C8-HSL and 3OC6-HSL. Overall these results are consistent with the findings of Collins et al., who reported that the mutations T33A S116A M135I (giving LuxR^A^) increase the sensitivity of the parent, LuxR^MJ1^, to C8-HSL, while the additional mutation R67M (giving LuxR^B^) restricted that sensitivity to exclude 3OC6-HSL [24].

However the response to combinations of the two HSLs is more complex than simple activation. In Fig 3 and S1 Fig the luminescence of LuxR^ES114^ and LuxR^MJ1^ is weakly suppressed by C8-HSL when 3OC6-HSL is present, while the luminescence of LuxR^B^ is weakly suppressed by 3OC6-HSL when C8-HSL is present. That is, in the presence of the “preferred” HSL, addition of the other HSL often causes some reduction of luminescence, suggesting that the two HSLs compete for interaction with LuxR.

### Role of *ainR*

**Fig 4:**
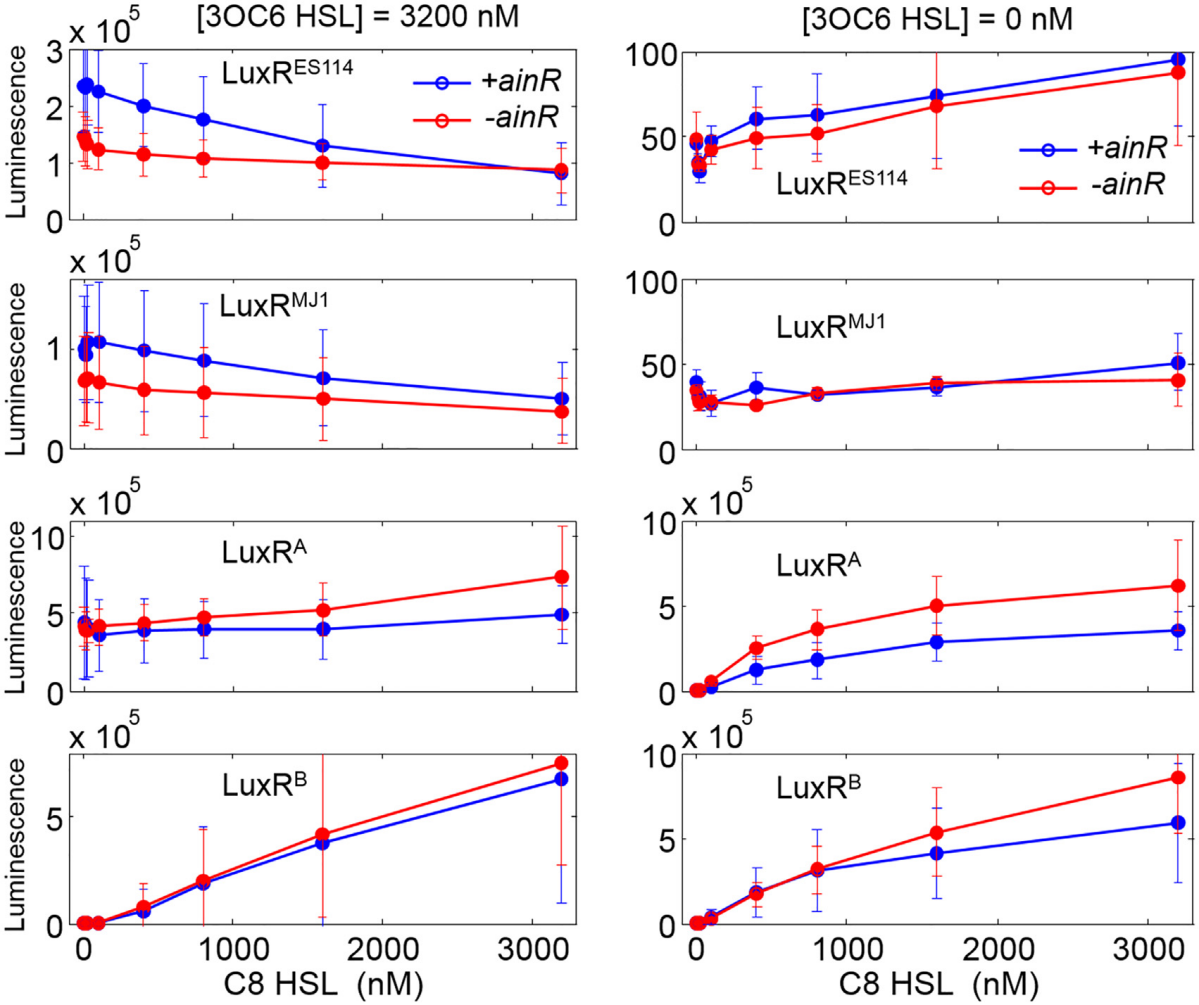
Effect of Δ*ainR* on the response to C8-HSL. Each panel shows the luminescence of a LuxR variant in both *ainR+* (blue) and *ainR-* (red) background. Left panels show results in the presence of 3.2 μM 3OC6-HSL and right panels show results in absence of 3OC6-HSL. All data are the average of at least three independent replicates. Error bars indicate standard deviation of the replicates. Luminescence is given in units of detector counts.

AinR is known to affect luminescence of wild-type *V*. *fischeri* by affecting transcriptional regulation of *luxR* and by influencing C8-HSL levels; however, in our experimental setup there is no endogenous C8-HSL production and *luxR* is transcribed from a non-native promoter. Thus, based on the documented roles of AinR, we would not expect it to influence luminescence in our experiments. Nonetheless, Fig 4 shows that *ainR* affects luminescence of our test strains, albeit modestly. The strains carrying LuxR^MJ1^ or LuxR^ES114^ luminesce more brightly when *ainR* is present than when it is deleted, at least in the presence of high 3OC6-HSL and low C8-HSL. This effect is reversed in strains carrying LuxR^A^ and LuxR^B^, for which the Δ *ainR* strains are slightly brighter at high C8-HSL. Importantly the overall response of strains with *ainR* is qualitatively similar to that of Δ *ainR* mutants, and the *ainR* genotype of the test strains had little if any influence on the analyses below.

### Modeling the performance of LuxR variants with defined mixtures of C8-HSL and 3OC6-HSL

Regardless of *ainR* genotype, the luminescence of otherwise isogenic strains is consistent with the expectation that both C8-HSL and 3OC6-HSL interact with LuxR to form complexes that drive transcription from the *lux* promoter, and that the strength of HSL association with LuxR and the affinity of those complexes for *lux* depend on the particular LuxR allele. In order to focus on the interactions between the two HSLs and the different LuxRs, we modeled the luminescence of the Δ*ainR* strains: For each of the *ΔainR* strains (DC19, DC20, DC62, and DC64; see Fig 2) we fit three independent luminescence datasets to the competitive binding model that is illustrated in Fig 1 and described in *Methods*. The fits yielded estimates for the six parameters that describe the interaction of each LuxR with the two HSLs.

**Fig 5:**
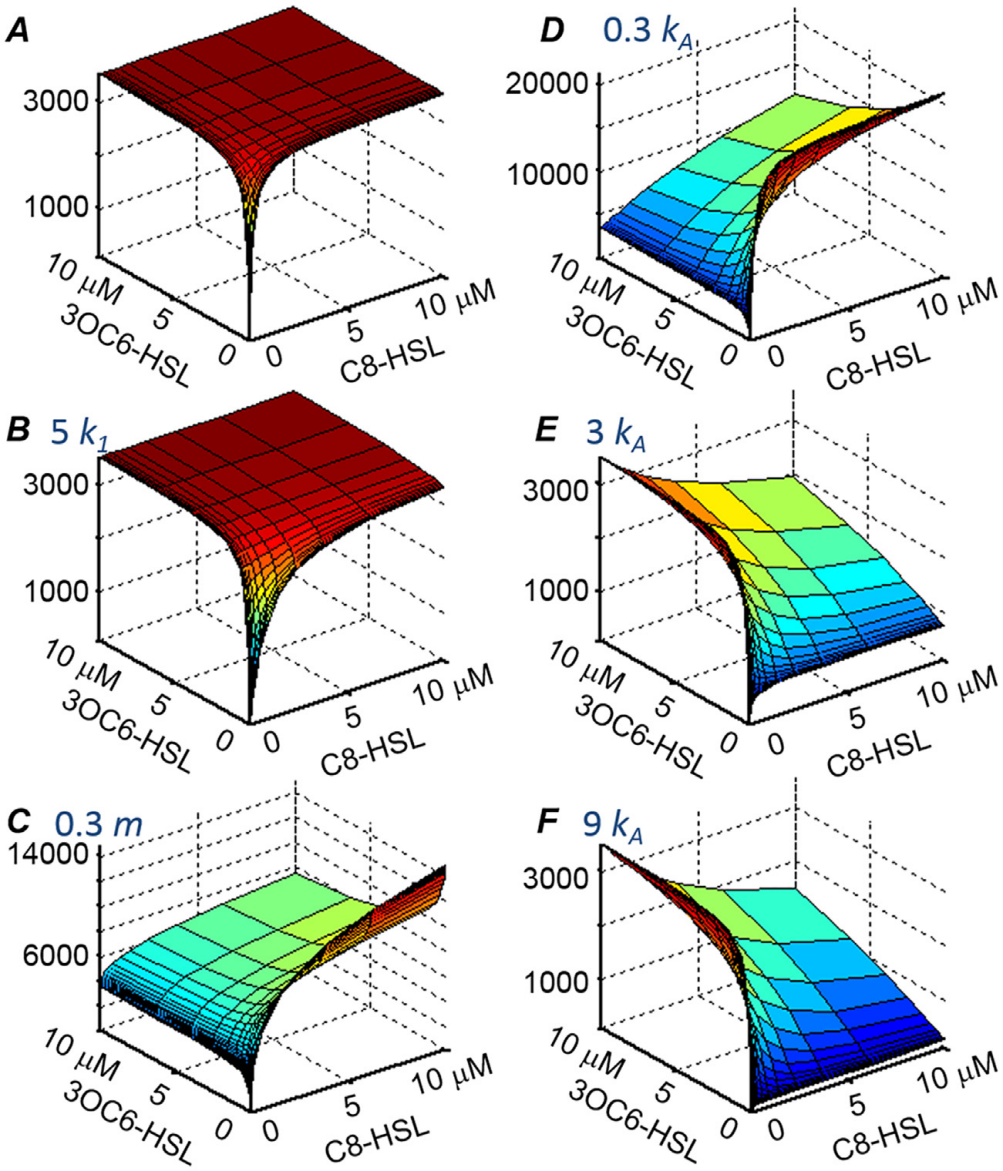
Illustration of the role of the model parameters. In order to show how the predicted HSL response is shaped by the values of the interaction parameters in the model (see *Methods*), the predicted response is shown for several different choices of the C8-HSL parameters while the parameters for 3OC6-HSL are held fixed. (Analogous figures are generated if the 3OC6-HSL parameters are varied while the C8-HSL parameters are held fixed). (*A*) Calculated luminescence signal (vertical scale) versus 3OC6-HSL and C8-HSL concentrations (horizontal scales), for parameter values *k*_*1*_ = 100 nM, *k*_*2*_ = 100 nM, *m* = 1, *n* = 1, *k*_*A*_ = 10 nM, *k*_*B*_ = 10 nM; (*B*) Calculated response for the same parameter values as in (*A*), except with *k*_*1*_ increased fivefold to *k*_*1*_ = 500 nM; (*C*) Response for same parameter values as in (*A*), except with *m* decreased to 0.3; (*D*)-(*F*) Response for same parameters as in (*A*), except with *k*_*A*_ changed to 3 nM, 30 nM, and 90 nM respectively. In all cases the overall scale parameters (Eq 4) are fixed at *a*_*0*_ = 10 and *a*_*1*_ = 550, which were typical for our datasets and analysis.

To help in the interpretation of Fig 3, we show in Fig 5 how the choice of model parameters shapes the luminescence response predicted by the model. The induction of luminescence by C8-HSL is governed by the parameters *k*_*1*_, *k*_*A*_, and *m*. Here *k*_*1*_ is the scaled (see *Methods*) dissociation constant and *m* is the Hill coefficient for the cooperative formation of the multimeric LuxR-C8-HSL complex. *k*_*A*_ is the scaled dissociation constant for the interaction of the complex with the *lux* box. The dissociation constants *k*_*1*_ and *k*_*A*_ differ in their effect on the maximum luminescence that is observed at saturating levels of C8-HSL. An increase in *k*_1_ increases the C8-HSL concentration that is required to attain saturating luminescence and reduces the ability of C8-HSL to compete with 3OC6-HSL for LuxR. By contrast, because the intracellular concentration of the LuxR-C8-HSL complex is limited by the intracellular LuxR concentration, a higher value of *k*_*A*_ reduces the maximum luminescence achieved at saturating concentrations of C8-HSL (Fig 5). The parameters *k*_*2*_, *k*_*B*_, and *n* play the same role for 3OC6-HSL that *k*_*1*_, *k*_*A*_, and *m* play for C8-HSL. Accordingly, if *k*_*A*_ = *k*_*B*_ but *k*_1_ ≠ *k*_*2*_, the saturated (high HSL) luminescence will not depend on which HSL is present. However if *k*_1_ = *k*_2_ but *k*_*A*_ ≠ *k*_*B*_, the fully saturated luminescence level will depend on which HSL or combination of HSLs is present.

The Hill coefficients *m* and *n* determine the initial curvature of the luminescence vs. C8-HSL and 3OC6-HSL profiles respectively. Because the empirical Hill coefficient reflects (imperfectly) the order of the LuxR-HSL multimer [35], and because intracellular LuxR concentration is finite, *m* and *n* affect the maximum possible degree of occupation of the *lux* site that can be attained at the highest HSL concentrations. Consequently in Fig 5, different values of *m* and *n* can lead to different saturating luminescence at high C8-HSL vs. 3OC6-HSL.

**Fig 6:**
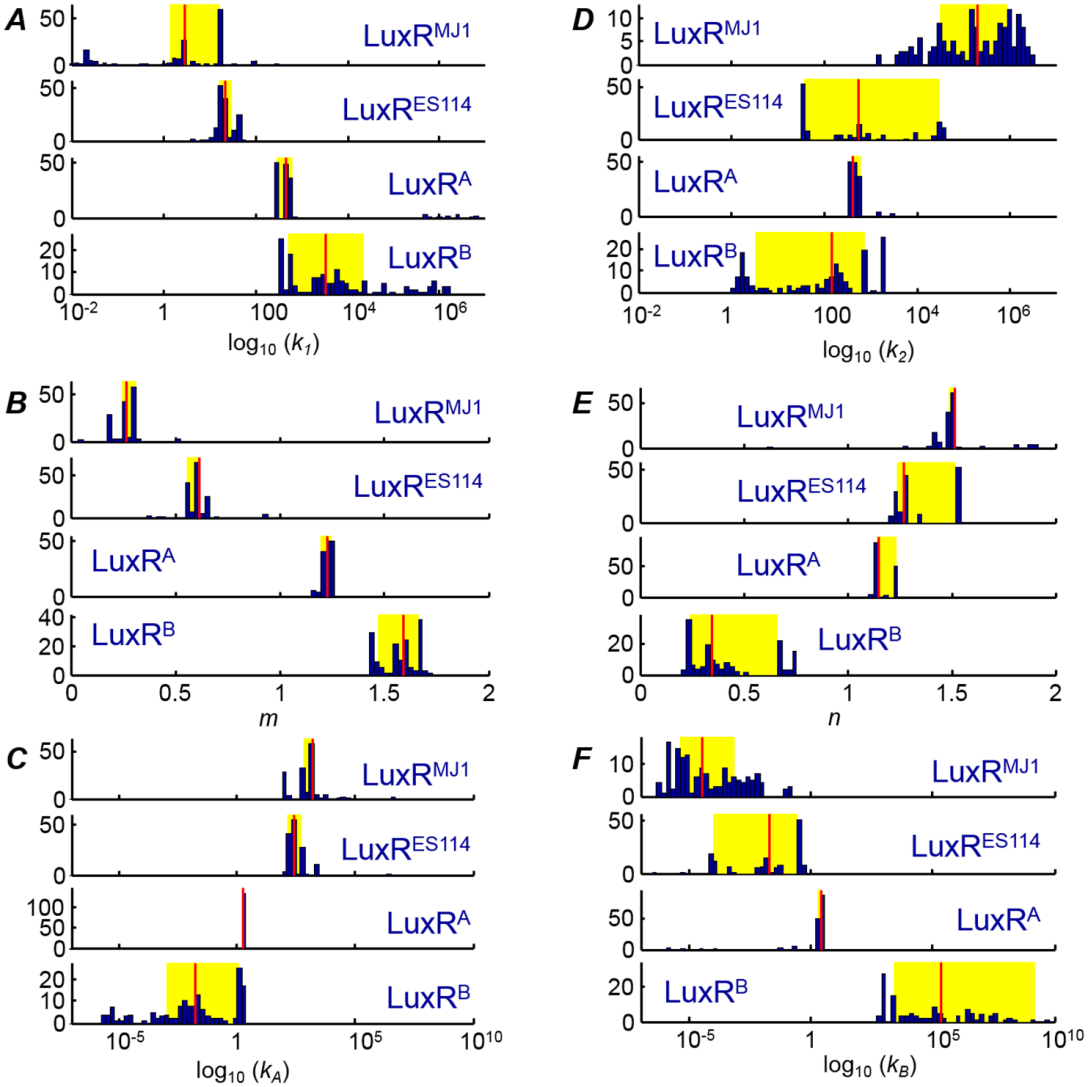
Fit results for the four *ΔainR* strains, obtained by fitting luminescence data for each strain to the six-parameter model of Fig 1. For each of four LuxR variants, 150 independent optimizations of the model were performed with respect to three independent luminescence experimental datasets. The histograms below indicate the results obtained for (*A*)-(*C*) the C8-HSL interaction parameters and (*D*)-(*F*) the 3OC6-HSL interaction parameters. The red line indicates the median result for a given parameter and LuxR, while the yellow box indicates the span of the 25^th^ -75^th^ percentiles for the parameter. The dissociation constants *k*_*1*_, *k*_*2*_, *k*_*A*_, *k*_*B*_ are the scaled (relative to [LuxR_*0*_]—see *Methods*) dissociation constants and accordingly have units of nM.

**Fig 7:**
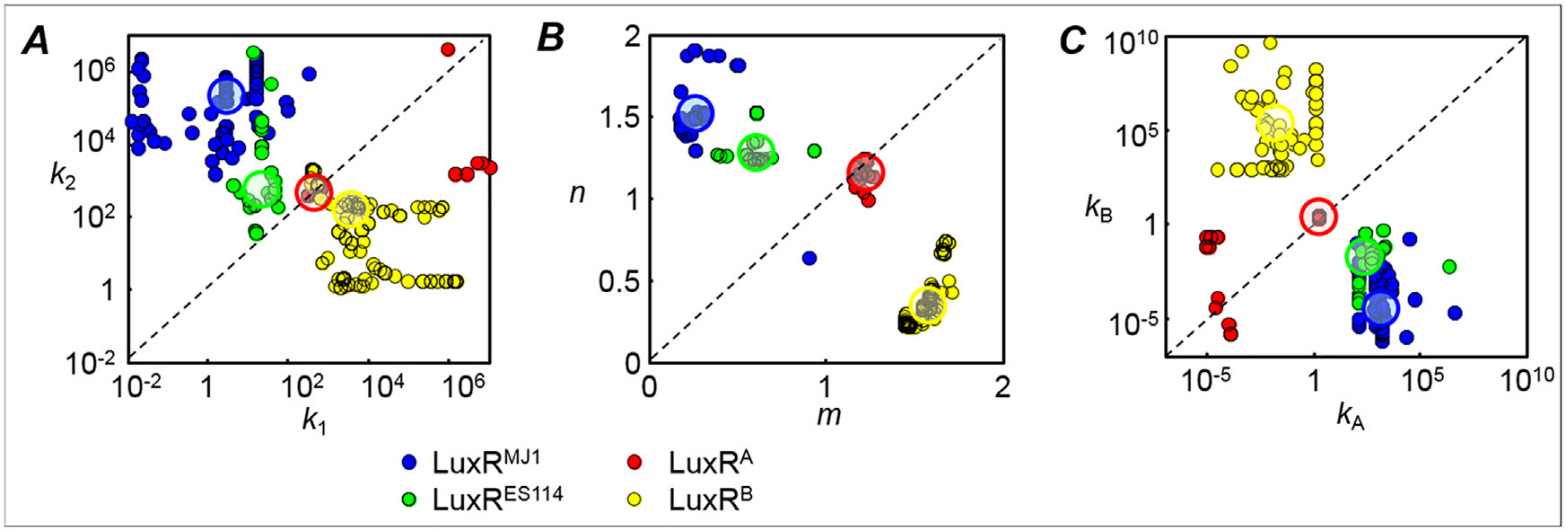
Correlation between interaction parameters for C8-HSL and 3OC6-HSL. Correlation between interaction parameters for C8-HSL (horizontal axes) and 3OC6-HSL (vertical axes) is shown for the four LuxR variants. All strains are Δ*ainR*. Each point represents parameter values obtained in one of the 150 fits performed for each LuxR. The color code (blue = LuxR^MJ1^, green = LuxR^ES114^, red = LuxR^A^, yellow = LuxR^B^) indicates the LuxR variant. Panels (*A)*, (*B)*, and (*C*) show results for complex dissociation (*k*_*1*_, *k*_*2*_), Hill coefficient (*m*, *n*), and *lux* binding (*k*_*A*_, *k*_*B*_) respectively. The black dashed line in each panel corresponds to equality between C8-HSL and 3OC6-HSL parameters; *k*_*1*_ = *k*_*2*_, *m* = *n*, or *k*_*A*_ = *k*_*B*_. The larger shaded circles highlight (with the same color code) the median value obtained for each LuxR.

The fitting of the model to the luminescence data for the four LuxR variants is described in *Methods*. Fig 3 and S1 Fig compare the luminescence data and fits. The fits provide excellent agreement with the data, capturing the range of different sensitivities and asymmetries that are seen in the HSL responses. The fits also capture the non-monotonic response where, in the presence of one HSL, addition of the second HSL can cause the luminescence to decrease. This behavior results when the LuxR complex formed with the second HSL has lower affinity for the *lux* box than does the LuxR complex with the first HSL. For example, in LuxR^ES114^, the bright luminescence induced by low concentrations of 3OC6-HSL is suppressed by introduction of C8-HSL; LuxR^ES114^ has a higher affinity for C8-HSL than for 3OC6-HSL, but the C8-HSL complex has a weaker affinity for *lux*. Figs 6 and 7 and Table 2 summarize the parameter estimates. In most cases we obtain robust parameter estimates that differ significantly among the different LuxRs, such that the uncertainties in the dissociation constants (*k*_*1*,_ *k*_*2*,_ *k*_*A*,_ *k*_*B*_) are generally smaller than the strain-to-strain differences in these parameters. The Hill coefficients *m* and *n* are determined to excellent precision, with weak cooperativity evident in some interactions but absent in others (e.g. *n* ≃ 1.5, *m* ≃ 0.3 for LuxR^MJ1^). The parameters *k*_*A*_ and *k*_*B*_ are especially well-determined for LuxR^A^ (Fig 6).

**Table 2:** Parameter estimates obtained from fit to luminescence (vs. C8-HSL and 3OC6-HSL) data.

	**LuxR^MJ1^** / DC64	**LuxR^ES114^** / DC62	**LuxR^A^** / DC19	**LuxR^B^** / DC20
**Log**_**10**_ **(*k***_**1**_ **(nM))**	0.46 (−1.4–+1.2)	1.3 (1.2–1.6)	2.6 (2.5–2.8)	3.5 (2.7–4.7)
***m***	0.27 (.20–.31)	0.61 (0.55–0.62)	1.23 (1.20–1.25)	1.6 (1.5–1.7)
**Log**_**10**_ **(*k***_**2**_ **(nM))**	5.3 (4.4–6.0)	2.7 (1.6–4.5)	2.63 (2.6–2.8)	2.2 (0.3–2.9)
***n***	1.51 (1.47–1.52)	1.3 (1.2–1.5)	1.15 (1.14–1.24)	0.34 (0.23–0.66)
**Log**_**10**_ **(*k***_**A**_ **)**	3.2 (2.1–3.2)	2.4 (2.2–2.7)	0.27 (0.24–0.29)	−1.8 (−3.5 - +0.1)
**Log**_**10**_ **(*k***_**B**_ **)**	−4.5 (−5.5–−2.8)	−1.7 (−4.0–−0.5)	0.41 (0.31–0.44)	5.3 (3.2–15)

Parameters *k*_1_ and *k*_2_ are scaled (Eq 2) dissociation constants for the HSL complexes of LuxR, which form with cooperativity *m* and *n* (Eq 1) respectively (See *Methods*). *k*_A_ and *k*_B_ are scaled (Eq 3) dissociation constants for the activation of *lux* by those complexes. See also Fig 5. The uncertainty ranges represent the 20^th^— 80^th^ percentile of fit results.

However some parameters are poorly determined for certain LuxRs. Although LuxR^B^ has a smaller dissociation constant *k*_*A*_ (and a larger *k*_*B*_) than do the other LuxRs, these parameters are subject to large uncertainty. The uncertainties reflect the fact that LuxR^B^ is strongly activated by even small amounts of C8-HSL (consistent with an indeterminately small *k*_*A*_), but is relatively unresponsive to 3OC6-HSL (consistent with an indeterminately large *k*_*B*_).

## Discussion

In this study we have measured the effect of four LuxR alleles on the luminescence that is induced in *V*. *fischeri* in response to combinations of two HSLs. In order to understand the large, qualitative differences in the behavior of the different LuxR alleles, we fit the data to a simplified binding model that assumes a simple, cooperative multimerization of LuxR in the presence of each HSL, so that two different types of *lux*-activating complexes (C8-HSL-LuxR and 3OC6-HSL-LuxR) are formed. The model contains six free parameters, which are determined with adequate precision using the array of HSL concentrations that were studied. It may also be of interest to consider more complex models, such as those that involve heterogeneous complexes where LuxR multimerizes with both C8-HSL and 3OC6-HSL simultaneously. However, introducing heteromultimers would increase the complexity of the model by adding at least three new fit parameters that would be poorly constrained by the data; we find the data are already well described by the simpler model that lacks heteromultimers. Therefore in order to extract a clear, if simplified, picture of the interactions between the different LuxRs and the two HSLs, we consider only the simple homomultimer interactions illustrated in Fig 1.

Despite uncertainties in some of the estimates, Fig 6 shows that the parameters obtained from modeling vary systematically as we move from the wild-type variants LuxR^MJ1^ and LuxR^ES114^ (which respond strongly to 3OC6-HSL but not C8-HSL), to the less specific LuxR^A^, and finally to LuxR^B^ (which is activated only by C8-HSL). Hence the response of LuxR variants to combinations of two HSLs provides an indication of which molecular-level interactions have been altered in functionally distinct variants of LuxR. It also points toward particular interactions that permit these LuxRs to discriminate between the two HSLs and thus achieve a sensitive and specific pheromone response.

Because our estimates for the dissociation constants (*k*_*1*_, *k*_*2*_, *k*_*A*_, *k*_*B*_) are scaled with respect to intracellular LuxR concentrations (see *Methods*), we cannot rule out the possibility that some of the changes we observe in these parameters arise from variations in LuxR concentration, owing perhaps to unequal expression or stability of the four LuxRs. However such variations are unlikely to explain all of the parameter variation in Fig 6: across different LuxRs the range of values for any one dissociation constant spans orders of magnitude (e.g. *k*_*A*_ ≃ 10^–2^ for LuxR^B^ to *k*_*A*_ ≃ 10^3^ for LuxR^MJ1^), while the peak luminescence levels of the different strains vary no more than six fold (Fig 3 and S1 Fig). Further, strain-to-strain changes in the dissociation constants are often uncorrelated or anticorrelated: between LuxR^MJ1^ and LuxR^B^, for example, *k*_*1*_ increases ~1000× while *k*_*A*_ decreases ~10^5^×. Both of these changes cannot be due only to a difference in LuxR concentration. It appears more plausible that most of the strain-to-strain variability in the parameters reflects underlying differences in LuxR interactions.

The model parameters show some intriguing trends as mutations are introduced into wild-type LuxR^MJ1^. The C8-HSL and 3OC6-HSL preferences of LuxR^B^ are virtually the reverse of those of its parent LuxR^MJ1^, whereas the interactions of LuxR^A^ are more symmetric or neutral with respect to the two HSLs. For LuxR^A^ the estimates for *k*_*1*_ and *k*_*2*_ fall mostly along the symmetry line *k*_*1*_ = *k*_*2*_, and similarly *k*_*A*_ ≃ *k*_*B*_ and *m* ≃ *n* (Fig 7). In this sense LuxR^A^ acts as a neutral HSL receptor, interacting equally with C8-HSL and 3OC6-HSL: reversing its T33A S116A M135I (to restore LuxR^MJ1^) or else introducing R67M (to give LuxR^B^) creates selectivity for one or the other HSL. Fig 7 also shows that the C8-HSL and 3OC6-HSL sensitivity of LuxR^B^ is largely the reverse of that of the wild-type LuxRs (LuxR^ES114^ and LuxR^MJ1^). LuxR^B^ shows large *m*, *k*_*B*_, and *k*_*1*_, and small *n*, *k*_*A*_, *k*_*2*_; LuxR^MJ1^ and LuxR^ES114^ both show the reverse, i.e. large *m*, *k*_*B*_, *k*_*1*_ and small *n*, *k*_*A*_, *k*_*2*_.

In our results, selectivity for or against a particular HSL is manifested not only by an imbalance of the HSL-LuxR dissociation constant but also by an opposite cooperativity for the two HSLs: LuxR^B^ exhibits cooperative *m* ≃ 1.6 and *n* ≃ 0.3, while LuxR^MJ1^ shows the reverse. These non-integer Hill coefficients require some interpretation. In cooperative ligand binding the value of the Hill coefficient *n*_H_ represents a lower limit on the number of interacting binding sites; generally *n*_H_ is less than the number of sites. For a protein with *N* binding sites the Hill coefficient *n*_H_ will approach *n*_H_ = *N* only if the sites interact very strongly [35]. Therefore our finding of non-integer Hill coefficients (*n*, *m*) that in many cases exceed one is fully consistent with the expectation that the regulatory-active LuxR-HSL complex is dimeric. However the generally moderate values of *n* and *m* (*n* = 1.5 (LuxR^MJ1^) and *n* = 1.3 (LuxR^ES114^), *m* ≃ *n* = 1.2 (LuxR^A^), *m* = 1.6 (LuxR^B^)) imply that the association of these dimers with their preferred HSLs is not strongly cooperative.

However we also find that the LuxR interactions with non-preferred HSLs are characterized by Hill coefficients less than one: *m* ≃ 0.3 and 0.6 for LuxR^MJ1^ and LuxR^ES114^ respectively, and *n* ≃ 0.3 for LuxR^B^. A Hill coefficient *n*_*H*_ < 1 may constitute evidence for negative cooperativity, in the sense of multiple identical binding sites interacting anticooperatively, with each successive ligand binding with lower affinity. It may also simply indicate a heterogeneous system that offers multiple inequivalent binding sites for the ligand [36]. In either case, *n*_*H*_ < 1 implies that the variance in the number of bound ligands is smaller than would exist in a completely neutral (non-cooperative) multisite system [37]. Therefore the small Hill coefficients *m*, *n* ≃ 0.3 that characterize the interaction of some LuxRs with their non-preferred HSLs may imply that the binding of these HSLs to LuxR induces a conformational change that impedes the formation of a LuxR-HSL dimer or higher multimer, or else that the physical configuration of the complex simply does not provide multiple equivalent binding sites for the non-preferred HSL. Our model does not include a sufficient number of parameters to yield separate estimates for the degree of multimerization as well as the cooperativity of ligand binding to those multimers. Rather, when the empirical Hill coefficient determined by the model is less than unity for one HSL, it is simply diagnostic that, if a multimeric complex forms, the binding of multiple HSLs to the subunits of that complex is poorly coordinated or heterogeneous. The fact that we find qualitatively different cooperativity (*e*.*g*. *m* > 1, *n* < 1) of the LuxRs with respect to their preferred and non-preferred HSLs suggests that HSL specificity may be achieved at least in part through HSL-sensitive interaction between LuxR subunits in a complex. Mutations such as S116A, M135I, R67M evidently modify these interactions and hence the specificity. We note that the literature contains additional examples of enzymes having positive cooperativity with respect to one ligand and negative cooperativity with respect to another, as the ligand-subunit interactions need not be identical [38].

We also find that inverse correlation between *k*_*A*_ and *k*_*B*_ is accompanied by an opposite correlation between *m* and *n*: for the LuxRs where *k*_*A*_ *> k*_*B*_ we find *n* > *m*, and likewise where *k*_*B*_ *> k*_*A*_ we find *m* > *n*. Some inverse correlation between (e.g.) *k*_*A*_ and *m* is expected, as high *k*_*A*_ and low *m* have opposite effects on the luminescence at saturating levels of C8-HSL: if the luminescence at saturating concentrations of C8-HSL is to fall within a particular range, then higher *k*_*A*_ must generally accompany lower *m* and vice versa. The same argument applies to *k*_*B*_ and *n*. However, as the level of luminescence is not identical at saturating concentrations of both pheromones, the more complex correlation observed between pairs of parameters for different HSLs is unexpected. The fact that in three of the LuxRs the parameters for C8-HSL are anticorrelated with those for 3OC6-HSL, while in LuxR^A^ they are completely symmetric with respect to both HSLs, suggests a mechanistic tradeoff whereby optimizing LuxR for a strong and cooperative response to one HSL reduces its affinity and cooperativity for the other HSL.

Converting our scaled dissociation constants (*k*_*1*_, *k*_*2*_, *k*_*A*_, *k*_*B*_) to absolute dissociation constants (*K*_*1*_, *K*_*2*_, *K*_*A*_, *K*_*B*_) requires knowledge of the intracellular LuxR concentration. Although we are not aware of a literature value for [LuxR] for *V*. *fischeri*, Chai and Winans measured the concentration of intracellular TraR in *A*. *tumefaciens* [39]: in the presence of the *N*-3-octanoyl-HSL the average cell contained approximately 20 units of TraR protomer. For a typical bacterial cell volume of 0.7 μm^3^ this value suggests an intracellular concentration [TraR] ≃ 33 nM. If we assume that comparable amounts of LuxR are present in our engineered *V*. *fischeri*, we can convert our scaled fit parameters to absolute dissociation constants (see *Methods*). The results, shown in S1 Table and S2 Fig, show that most of the dissociation constants for the LuxR-HSL complexes (*K*_*1*_, *K*_*2*_) lie within a narrow range: For three of the four LuxRs, the median *K*_*2*_ values lie within a factor ∼3 of 300 nM. Likewise the median *K*_*1*_ values for three of the four LuxRs lie within the same range. Consequently, while the LuxR variants differ sharply in the cooperativity of their interactions with the HSLs, they show much less variation in the strength of those associations as measured by *k*_1_ and *k*_2_.

The strongest and weakest binding interactions in S1 Table describe the interaction of the complex with the *lux* promoter. The strongest interactions have subnanomolar dissociation constants and characterize the interaction between *lux* and the 3OC6-HSL complexes of LuxR^MJ1^ and LuxR^ES114^, and between *lux* and the C8-HSL complex of LuxR^B^. The weakest interactions are those between *lux* and the LuxR^B^-3OC6-HSL complex or between *lux* and the LuxR^MJ1^/LuxR^ES114^- C8-HSL complex. These dissociation constants appear to lie in the micromolar to millimolar range. These findings indicate that a key consequence of the non-cooperative interaction between LuxR and its non-preferred HSL is the formation of an inactive or nonfunctional multimer that does not readily interact with the *lux* promoter to activate transcription.

Our results provide additional insights into previous observations of LuxR structure and function. LuxR can be divided roughly into an N-terminal ligand-binding domain (residues 1–156), and a C-terminal DNA-binding domain (residues 157–250). As noted above, LuxR^ES114^ and LuxR^MJ1^ are more dissimilar than most orthologs in these strains, and this divergence is more evident in the N-terminal domain (73% identity) than in the C-terminal domain (88% identity). Moreover, C-terminal domain residues identified as critical for DNA binding [40] are absolutely conserved in LuxR^ES114^ and LuxR^MJ1^, as well as LuxR^A^ and LuxR^B^. It is not surprising that differences in responsiveness to distinct ligands would correlate with deviations in the N-terminal domain. However as Collins et al. [23] noted, some key residues appear to be outside the pheromone-binding pocket. For example, among the mutations to LuxR^MJ1^ conferring enhanced responsiveness to C8-HSL, they found a T33A allele, which intriguingly is also found in LuxR^ES114^, and lies in a region predicted to be distinct from pheromone binding.

Collins et al. also showed that the single point mutation R67M strongly affects HSL selectivity [24]. Strains with the LuxR^B^ allele, which contains the R67M substitution, exhibited sharply reduced activation by 3OC6-HSL and by other HSLs that contain the 3-oxo group, but showed an enhanced response to HSLs that lack the 3-oxo. Working with the LuxR homolog TraR of *A*. *tumefaciens*, Chai and Winans proposed that specificity for the 3-oxo group is provided by residues T129 and T115, which stabilize a bound water molecule that donates a hydrogen bond to the 3-oxo group [39]. An alignment of TraR and LuxR suggests that Ser123 and Ser127 in LuxR play a role similar to T129 and T115 in TraR. The S116A and M135I mutations may sufficiently disrupt the local structure to affect this specificity for the 3-oxo group. However the mechanism is not strictly localized to the pheromone-binding pocket, as we find cooperativity and *lux* binding are both strongly affected.

Consequently our data emphatically support the concept that changes in the N-terminal domain can alter the specificity of pheromone responses in a manner that goes beyond a straightforward effect on pheromone binding. Indeed, despite greater responsiveness to C8-HSL, LuxR^A^ and LuxR^B^ have a *higher K*_*1*_, suggesting weaker C8-HSL binding; their enhanced response to C8-HSL stems from a greater cooperativity of the C8-HSL-LuxR complex. It seems reasonable that residues exerting such an effect would lie in the N-terminal domain but not at the pheromone-binding site. The distinction between binding-driven and cooperativity-driven changes in pheromone specificity has important functional implications, notably including the potential for negative interactions wherein an inferior activator can inhibit the activity of a better one. In the future, the rational design of synthetic LuxR alleles to act as bioreporters of HSLs should take this distinction into account.

## Methods

### Strains and media

Strains used in this study are listed in Table 1. *V*. *fischeri* wild-type strain ES114 [19] served as the parental strain into which mutations and modifications were engineered. *Escherichia coli* strains DH5α [41] or DH5αλ*pir* [42] were used for cloning, with the latter used to harbor plasmids containing the R6K origin of replication. *E*. *coli* cultures were grown at 37°C in LB medium [43] with final concentrations of 20 μg ml^-1^ chloramphenicol or 40 μg ml^-1^ kanamycin added for selection when appropriate. *V*. *fischeri* was grown at 28°C in LBS medium [44] or at 24°C in SWTO medium [45], with 2 μg ml^-1^ chloramphenicol or 5 μg ml^-1^ erythromycin added to LBS for selection. IPTG was obtained from Sigma-Aldrich (St. Louis, MO), and was added to media at a final concentration of 1 mM as indicated below. 3OC6-HSL, and C8-HSL were also obtained from Sigma-Aldrich, the latter in an isomeric mix of *N*-octanoyl-DL-homoserine lactone. Each HSL was dissolved in ethyl acetate, a defined amount was added to a sterile glass container, the solvent was allowed to evaporate, medium was added such that the HSL concentration was 6.4 μM, and this stock was then further diluted as described below.

### Genetic manipulations

Table 1 lists key plasmids and oligonucleotides used to engineer mutant strains, and details of cloning intermediates and strain construction are available on request. Mutant alleles were transferred from *E*. *coli* into *V*. *fischeri* ES114 on plasmids by triparental mating using the conjugative helper plasmid pEVS104 [46] in strain CC118λ*pir* [47], and allelic exchange was screened phenotypically and confirmed by PCR. Plasmids were constructed using standard techniques. A summary of the strains used to assay LuxR activity is illustrated in Fig 2A, a schematic overview of the sequences engineered near the *luxI-luxR* locus is shown in Fig 2B, and a brief summary follows. To replace the native *luxR*-*luxI* we engineered constructs flanked by sequences from each side of the *luxR-luxI* locus. We began with plasmid pJLB72 [34], which contains ~1.5 kbp downstream of *luxI* (including *luxC*). We obtained a fragment of ~1.5 kbp downstream of *luxR* by PCR amplification using primers luxRdnFNotI and luxRdnRNheI. Fig 2B shows the junctions between native and engineered sequence. Between the sequences upstream and downstream of the *luxR-luxI* locus we engineered a small region such that the “*lux* box”-containing (LuxR-dependent) *luxI* promoter drives *luxCDABEG* expression and *luxR* is divergently transcribed from a constitutive promoter (Fig 2).

To generate this engineered *lux* locus, the *luxI* promoter was PCR amplified off ES114 template DNA using primers PluxIF2 and PluxIR (Table 1), and this fragment was digested using restriction sites on the primer ends and cloned between *Avr*II and *Apa*I sites such that it would drive expression of *luxC* when crossed into the genome (Fig 2B). Oligonucleotides Pconoligo1 and Pconoligo2 (Table 1) were annealed to make a fragment containing a near-consensus constitutive promoter along with single-stranded overhangs that enabled us to clone it between *Apa*I and *Xho*I sites (Fig 2B). The *luxR* variants were PCR amplified, and the amplicons were digested with *Not*I and *Xho*I and cloned between *Not*I and *Xho*I sites, so that their expression would be driven by the artificial constitutive promoter (Fig 2B). The ES114 *luxR* (*luxR* ^ES114^) was amplified using primers 5’-ESll4luxRXhoI and 3’-ESll4luxRNotI (Table 1), and the MJ1 *luxR* (*luxR* ^MJ1^) was amplified by primers 5’-LuxRXhoI and 3’-LuxRNotI (Table 1). The LuxR variants evolved from *luxR* ^MJ1^ [23,24] were amplified using primers 5’-LuxRXhoI and 3’-LuxRNotI (Table 1) using plasmids pLuxR-G2E (allele *luxR* ^A^) and pLuxR-G2E-R67M (allele *luxR* ^B^) as templates, respectively.

To facilitate screening of allelic exchange wherein the native *luxI-luxR* region was replaced with *luxR* alleles of interest in *ainS* and *ainSR* mutant backgrounds (Fig 2) we first used pJLB101 to place *lacI* ^q^ and the LacI^q^-controlled P_AI/34_ promoter upstream of *luxC* in strains NL60 (Δ*ainS*) and NL55 (Δ*ainSR*), generating strains DC36 and DC44, respectively. The *luxR* alleles and engineered constructs on plasmids pDJ01 (*luxR* ^ES114^), pDC55 (*luxR* ^MJ1^), pDC36 (*luxR* ^A^), and pDC37 (*luxR* ^B^) (Table 1), were crossed into DC36 (Δ*ainS*), with allelic exchange easily screened by a loss of IPTG-inducible bioluminescence, generating strains, DJ01, DC43, DC21, and DC22 (Fig 2). Similarly, the engineered alleles on plasmids pDJ01, pDC55, pDC36, and pDC37, were crossed into DC44 (Δ*ainSR*), generating strains, DC62, DC64, DC19, and DC20 (Fig 2). As a negative control, the engineered allele bearing no *luxR* on pDC44 was crossed into DC36, generating strain JHK007 (Fig 2).

### Luminescence responses to 3OC6-HSL and C8-HSL

6.4 μM stocks of 3OC6-HSL or C8-HSL were serially diluted in SWTO medium to the final concentrations indicated. An overnight culture of the strain being tested was diluted 1:100 into 200 μl of SWTO in each well of a 96-well black clear-bottom plate (Greiner Bio-One, Monroe, NC). Treatments were arranged, and blank wells were included, to minimize potential light contamination between treatments skewing results. Specifically, final concentrations of 0, 12.5, 25, 100, 400, 800, 1600, and 3200 nM of C8-HSL were used in consecutive horizontal rows, and the same concentrations of 3OC6-HSL were placed in consecutive vertical columns, with the placement of C8-HSL and 3OC6-HSL reversed for the assays with DC20 and DC22. Blank columns were included after the first four columns to help minimize light contamination. Plates were incubated at 24°C and while shaking at 200 rpm, and the optical density at 600 nm (OD_600_) and luminescence were measured in a Synergy 2 plate reader (BioTek, Winooski, VT). OD_600_ readings were divided by 0.46 to correspond to the optical density of a 1-cm path-length, and relative luminescence was normalized to the path-length corrected OD_600_ of ~1.0 to give specific luminescence for each experimental condition.

### Modeling

Well-plate studies performed for each LuxR variant provided the bioluminescence as a function of the C8-HSL and 3OC6-HSL concentration. We fit these data to the LuxR activation model illustrated in Fig 1. In this model LuxR can form a multimeric complex with either C8-HSL or 3OC6-HSL, and both of these complexes can interact with the *lux* box to activate transcription and bioluminescence. The LuxR-C8-HSL complex forms cooperatively with a Hill coefficient of *m*, such that it is modeled to consist of *m* copies of LuxR and *m* molecules of C8-HSL, and has dissociation constant *K*_*1*_ (nM). Similarly the 3OC6-HSL-LuxR complex forms with a Hill coefficient *n*, and is modeled to have *n* copies of LuxR and *n* molecules of 3OC6-HSL, and has dissociation constant *K*_*2*_ (nM). The model does not require *m* or *n* to be integers. It also does not allow mixed LuxR complexes containing both C8-HSL and 3OC6-HSL. The C8-HSL and 3OC6-HSL complexes of LuxR bind to the *lux* site with dissociation constants *K*_*A*_ (nM) and *K*_*B*_ (nM) respectively. The rate of transcription of *lux* is taken as proportional to the occupancy of the *lux* site by LuxR complex, where the rate is the same regardless of which HSL complex is bound.

The formation of the two LuxR complexes are characterized by dissociation constants

$$\begin{array}{l} K_{1}{}^{2\text{m}-1}=\;{\left[\text{C}8-\mathrm{HSL}\right]}^{\text{m}}{\left[\mathrm{LuxR}\right]}^{\text{m}}/\;\left[{\left(\mathrm{LuxR}-\text{C}8\mathrm{HSL}\right)}_{\text{m}}\right]\\ K_{2}{}^{2\text{n}-1}=\;{\left[3\mathrm{OC}6-\mathrm{HSL}\right]}^{\text{n}}{\left[\mathrm{LuxR}\right]}^{\text{n}}/\;\left[{\left(\mathrm{LuxR}-3\mathrm{OC}6\mathrm{HSL}\right)}_{\text{n}}\right] \end{array}$$ (1)

Here *K*_*1*_ and *K*_*2*_ are defined with the powers 2*m* -1 and 2*n*-1 so that they have units of concentration (nanomolar). [LuxR] describes the intracellular concentration of free LuxR and [(LuxR-C8HSL)_*m*_] describes the concentration of the LuxR-C8-HSL complex, etc.

If *p*_0_ is the probability that there is no LuxR complex at the *lux* activation binding site, then *p*_A_ (or *p*_B_) is the probability that the C8-HSL (or 3OC6-HSL respectively) complex is at the *lux* site. Then we can define the probability ratios

$$\begin{array}{l} P_{A}=\;p_{A}/p_{0}=\;\left[{\left(\mathrm{LuxR}-\text{C}8\mathrm{HSL}\right)}_{\text{m}}\right]/K_{A}\\ P_{B}=\;p_{B}/p_{0}=\;\left[{\left(\mathrm{LuxR}-3\mathrm{OC}6\mathrm{HSL}\right)}_{\text{n}}\right]/K_{B} \end{array}$$

Expression of the *lux* operon, and consequently the concentration of the constituent subunits of the luciferase dimer, are presumed to be proportional to the probability that the *lux* activation binding site is occupied by either the C8-HSL or the 3OC6-HSL complex:

$$P=\;\left(p_{\text{A}}+p_{\text{B}}\right)/\left(p_{0}+p_{\text{A}}+p_{\text{B}}\right)\;=\;\left(P_{\text{A}}+P_{\text{B}}\right)/\left(1\;+P_{\text{A}}+P_{\text{B}}\right)$$

As the occupancy *P* and hence the bioluminescence can be calculated for any set of model parameters and HSL concentrations, we can estimate the model parameters by fitting bioluminescence data. However such a fit does not determine the intracellular concentration of LuxR (which is not measured in our experiments) independently of the four dissociation constants *K*_*1*_, *K*_*A*_, *K*_*2*_, and *K*_*B*_. Consequently we scale these dissociation constants to the total (free + bound) LuxR concentration inside the cell, denoted LuxR_0_. This gives the scaled parameters *k*_*1*_, *k*_*A*_, *k*_*2*_, and *k*_*B*,_ which we can determine by fitting the bioluminescence data.

To rewrite the model in terms of scaled parameters, we first define the scaled concentration of free LuxR inside the cell,

$$r=\;\left[\mathrm{LuxR}\right]/[\mathrm{Lux}{\text{R}}_{0}],$$

and the scaled dissociation constants for the C8-HSL and 3OC6-HSL complexes of LuxR,

$$\begin{array}{l} k_{1}{}^{\text{m}}=K_{1}{}^{2\text{m}-1}/{\left[\mathrm{Lux}{\text{R}}_{0}\right]}^{\text{m}-1}\\ k_{2}{}^{\text{n}}=K_{2}{}^{2\text{n}-1}/{\left[\mathrm{Lux}{\text{R}}_{0}\right]}^{\text{n}-1} \end{array}$$ (2)

Note that *k*_*1*_ and *k*_*2*_ are defined with powers *m* and *n* so as to have units of concentration (nM). We can also scale the concentrations of the two HSL complexes of LuxR,

$$\begin{array}{l} rc8\;=\;\left[{\left(\mathrm{LuxR}-\text{C}8\mathrm{HSL}\right)}_{\text{m}}\right]/\left[\mathrm{Lux}{\text{R}}_{0}\right]\;=\;{\left[\text{C}8\mathrm{HSL}\right]}^{\text{m}}r^{\text{m}}/k_{1}{}^{\text{m}}\\ rc6\;=\;\left[{\left(\mathrm{LuxR}-3\mathrm{OC}6\mathrm{HSL}\right)}_{\text{n}}\right]/\left[\mathrm{Lux}{\text{R}}_{0}\right]\;=\;{\left[3\mathrm{OC}6\mathrm{HSL}\right]}^{\text{n}}r^{\text{n}}/k_{2}{}^{\text{n}} \end{array}$$

Then the occupancy of the *lux* binding site is

$$P=\;\left(P_{\text{A}}+P_{\text{B}}\right)/\left(1\;+P_{\text{A}}+P_{\text{B}}\right)$$

where

$$\begin{array}{l} P_{\text{A}}=\;\left[{\left(\mathrm{LuxR}-\text{C}8\right)}_{\text{m}}\right]/K_{\text{A}}=rc8/k_{\text{A}}\\ P_{\text{B}}=\;\left[{\left(\mathrm{LuxR}-3\mathrm{OC}6\mathrm{HSL}\right)}_{\text{n}}\right]/K_{\text{B}}=rc6/k_{\text{B}} \end{array}$$

$$\begin{array}{l} k_{\text{A}}=K_{\text{A}}/\left[\mathrm{Lux}{\text{R}}_{0}\right]\\ k_{\text{B}}=K_{\text{B}}/\left[\mathrm{Lux}{\text{R}}_{0}\right] \end{array}$$ (3)

For a given LuxR strain, we can determine the scaled dissociation constants *k*_A_, *k*_B_, *k*_1_, *k*_2_ as well as *m* and *n* by fitting the experimental luminescence data as a function of the exogenous [C8-HSL] and [3OC6-HSL].

As the luciferase is constituted as a dimer LuxA-LuxB, the bacterial luminescence is expected to be proportional to the square of the transcriptional activity, or the occupancy of the *lux* site should be proportional to the square root of the measured bioluminescence. (This relationship is evident when (e.g.) the bioluminescence is compared with the expression of a GFP reporter for the *lux* operon [29].) Therefore, to fit an experimental data array *L*_data_([C8HSL],[3OC6HSL]), we assume that the square root of the luminescence is proportional to *P*:

$$L_{\text{model}}{}^{½}\;=a_{0}+a_{1}P$$ (4)

Given an experimental dataset and the six parameters *k*_A_, *k*_B_, *k*_1_, *k*_2_, *m*, and *n*, we can then find the two constants *a*_0_ and *a*_1_ that optimally align *L*_model_^1/2^ to *L*_data_^1/2^ in a least squares sense. Therefore the fitting procedure (1) begins with a choice of the six model parameters and evaluation of *P;* (2) The optimal *a*_0_ and *a*_1_ are then found by a linear least squares alignment of *L*_model_^1/2^ to *L*_data_^1/2^; (3) The error between model and fit is evaluated; (4) The six model parameters are revised and the cycle is repeated (via a Nelder-Mead simplex search) until optimal model parameters are found. Note that in step (3) the error between model and fit is calculated on a logarithmic scale, minimizing the sum of squares difference between log(*L*_data_) and log(*L*_model_), as the bioluminescence data span many decades in magnitude and the uncertainties are more nearly proportional than absolute. In practice the numerical values for the scaling parameters in Eq (4) were found to be *a*_*1*_ = 300–1000, *a*_*0*_ ≈ 10.

In order to obtain initial seed values for the fit procedure described above, we conducted for each LuxR variant a global search of a broad parameter space, looking for parameter combinations that gave rough agreement with the data. This step begins, for each LuxR strain, with a random selection of 30,000 points in the six-dimensional parameter space of [log *k*_1_, *m*, log *k*_2_, *n*, log *k*_A_, log *k*_B_]. We assessed the fitting error for each point and retained the best 50 (0.16%) of these parameter values. These best values were used as seed values for the optimization routine described above, leading to multiple independently-obtained sets of optimized parameters for each LuxR strain. We then used those parameter values repeatedly as seed values for multiple optimizations of the model with respect to three independent experimental luminescence datasets for each strain. In this way, three datasets were fit multiple times using different sets of seed parameters, for a total of 150 separate optimizations per LuxR strain. The resulting 150 estimates for each of the model parameters, for each LuxR, are summarized in Table 2 and Figs 6 and 7. Fig 3 compares the experimental datasets with typical curves generated by the fitting.

## Supporting Information

S1 Dataset Spreadsheet containing fit results for LuxRA strain. (XLSX)

S2 Dataset Spreadsheet containing fit results for LuxRB strain. (XLSX)

S3 Dataset Spreadsheet containing fit results for LuxR ES114 strain. (XLSX)

S4 Dataset Spreadsheet containing fit results for LuxR MJ1 strain. (XLSX)

S5 Dataset Spreadsheet containing luminescence data for +*ainR* strains. (XLSX)

S6 Dataset Spreadsheet containing luminescence data for -*ainR* strains. (XLSX)

S1 Fig Comparing data and fit for Δ*ainRS* mutants of the four LuxR variants. Each of panels (*A*)-(*D*) shows a representative luminescence dataset and fit for one of the Δ*ainRS* strains, where luminescence is measured as a function of C8-HSL and 3OC6-HSL concentration. The vertical axis indicates luminescence data and fit in units of fluorimeter counts on a linear scale, although the least-squares fitting was performed on a logarithmic scale (see *Methods* and Fig 3). The lower figure of each group shows the simple residual, *data—fit*, on a linear scale.
(TIF)

S2 Fig Correlation between estimated absolute parameters. The figure shows correlation between estimated absolute interaction parameters for C8-HSL (horizontal axes) and 3OC6-HSL (vertical axes), for the four LuxR variants in Δ*ainR* mutants. Each point represents parameter values obtained in one of the 150 fits performed for each LuxR. The color code (blue = LuxR^MJ1^, green = LuxR^ES114^, red = LuxR^A^, yellow = LuxR^B^) indicates the LuxR variant studied in the fit. Unlike in Fig 7, the scaled parameters (*k*_*1*_, *k*_*2*_, *k*_*A*_, *k*_*B*_) obtained from fitting are converted to absolute parameters (*K*_*1*_, *K*_*2*_, *K*_*A*_, *K*_*B*_ in nM) by assuming [LuxR_0_] ≃ 33 μM. Each point represents one fit result (out of 150 results total) obtained for one strain. Panels (*A*) and (*B*) show results for complex dissociation (*K*_*1*_, *K*_*2*_), and *lux* binding (*K*_*A*_, *K*_*B*_) respectively. The black dashed line in each panel corresponds to equality between C8-HSL and 3OC6-HSL parameters; *K*_*1*_ = *K*_*2*_, or *K*_*A*_ = *K*_*B*_. The larger shaded circles highlight (with the same color code) the median value obtained for each LuxR.
(TIF)

S1 Table Estimated absolute dissociation constants. Absolute dissociation constants for the model of Fig 1, estimated from fit results and an assumed intracellular LuxR concentration of 33 nM. The first value given for each parameter is based on the median fit result for that parameter; the indicated range encompasses the 20th to 80th percentile of the fit results.
(PDF)
