# Supplementary figures and images for: Modeling Analysis of Signal Sensitivity and Specificity by Vibrio fischeri LuxR Variants
Source: PLoS One. 2015 May 11;10(5):e0126474. doi: 10.1371/journal.pone.0126474 (PMC4427320; doi:10.1371/journal.pone.0126474)

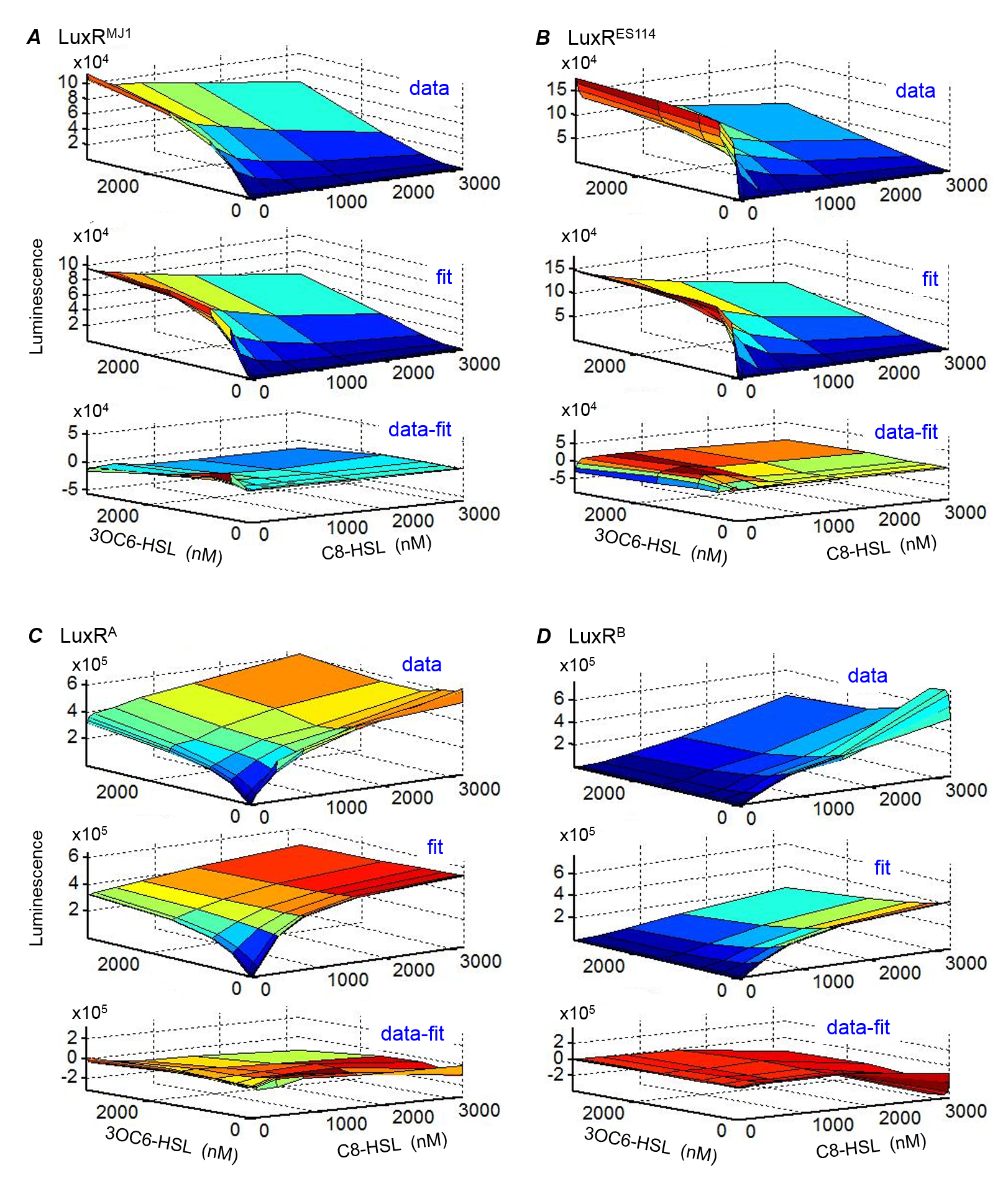

Supplement: S1 Fig — Each of panels (A)-(D) shows a representative luminescence dataset and fit for one of the ΔainRS strains, where luminescence is measured as a function of C8-HSL and 3OC6-HSL concentration. The vertical axis indicates luminescence data and fit in units of fluorimeter counts on a linear scale, although the least-squares fitting was performed on a logarithmic scale (see Methods and Fig 3). The lower figure of each group shows the simple residual, data—fit, on a linear scale. (TIF) [file pone.0126474.s007.tif]

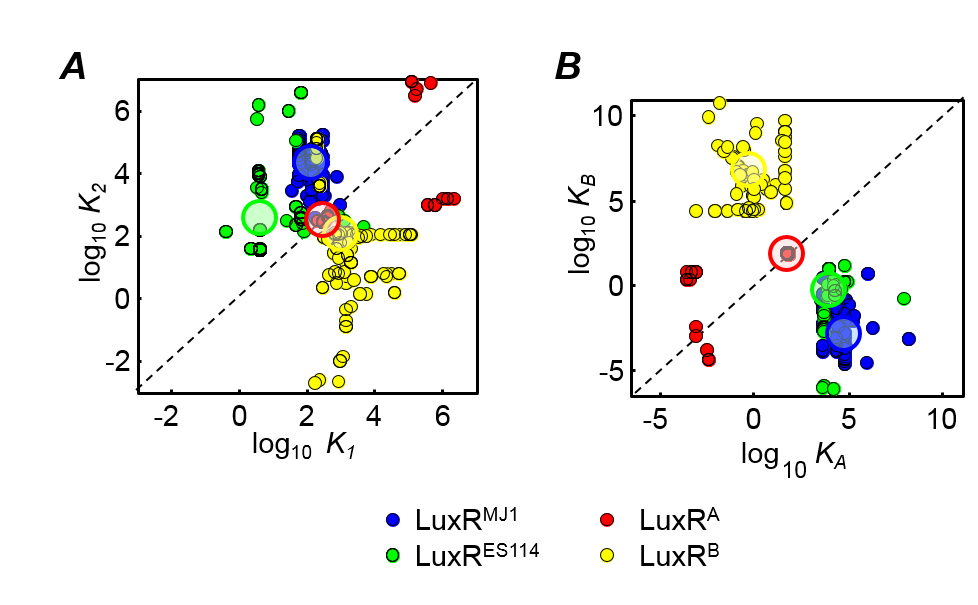

Supplement: S2 Fig — The figure shows correlation between estimated absolute interaction parameters for C8-HSL (horizontal axes) and 3OC6-HSL (vertical axes), for the four LuxR variants in ΔainR mutants. Each point represents parameter values obtained in one of the 150 fits performed for each LuxR. The color code (blue = LuxRMJ1, green = LuxRES114, red = LuxRA, yellow = LuxRB) indicates the LuxR variant studied in the fit. Unlike in Fig 7, the scaled parameters (k 1, k 2, k A, k B) obtained from fitting are converted to absolute parameters (K 1, K 2, K A, K B in nM) by assuming [LuxR0] ≃ 33 μM. Each point represents one fit result (out of 150 results total) obtained for one strain. Panels (A) and (B) show results for complex dissociation (K 1, K 2), and lux binding (K A, K B) respectively. The black dashed line in each panel corresponds to equality between C8-HSL and 3OC6-HSL parameters; K 1 = K 2, or K A = K B. The larger shaded circles highlight (with the same color code) the median value obtained for each LuxR. (TIF) [file pone.0126474.s008.tif]
