# Supplementary material for: Modeling Analysis of Signal Sensitivity and Specificity by Vibrio fischeri LuxR Variants
Source: PLoS One. 2015 May 11;10(5):e0126474. doi: 10.1371/journal.pone.0126474 (PMC4427320; doi:10.1371/journal.pone.0126474)
Supplement: S1 Table — Absolute dissociation constants for the model of Fig 1, estimated from fit results and an assumed intracellular LuxR concentration of 33 nM. The first value given for each parameter is based on the median fit result for that parameter; the indicated range encompasses the 20th to 80th percentile of the fit results. (PDF) [file pone.0126474.s009.pdf]

S1 Table. Estimated absolute dissociation constants. Absolute dissociation constants for the model of Fig. 1, estimated from fit results and an assumed intracellular LuxR concentration of 33 nM. The first value given for each parameter is based on the median fit result for that parameter; the indicated range encompasses the 20th to 80th percentile of the fit results.

	**$LuxR^{MJ1}$**	**$LuxR^{ES114}$**	**$LuxR^{A}$**	**$LuxR^{B}$**
K_1	130 nM (60-290 nM)	4.3 nM (3.9 – 48 nM)	300 nM (210-410 nM)	1.1 µM (240 nM – 7.5 µM)
K_2	23 µM (5.6 – 85 µM)	340 nM (35 nM – 9.5 µM)	300 nM (290 – 440 nM)	110 nM (4.8 nM – 4.0 µM)
K_A	54 µM (4.4 – 56 µM)	8.4 µM (4.7 – 17 µM)	61 nM (57 – 64 nM)	0.55 nM (0.01 – 44 nM)
K_B	0.001 nM (<0.05 nM)	0.6 nM (< 9.4 nM)	84 nM (68 – 90 nM)	7 mM (> 60 µM)
